# Efficacy of simultaneous aerobic exercise and cognitive training in subjective cognitive decline: study protocol for randomized controlled trial of the Exergames Study

**DOI:** 10.1186/s13063-020-04950-7

**Published:** 2021-01-06

**Authors:** Dereck Salisbury, Tom Plocher, Fang Yu

**Affiliations:** 1 University of Minnesota School of Nursing, 5-160 WDH 1331, 308 Harvard St SE, Minneapolis, MN 55455 USA; 2 Moai Technologies LLC, Minneapolis, MN USA; 3 Arizona State University, Health North Suite 301, 550 North 3rd Street, Mail Code 3020, Phoenix, AZ 85004 USA

**Keywords:** Aerobic exercise, Cognitive training, Cognition, Subjective cognitive decline, Exercise, Alzheimer’s disease

## Abstract

**Background:**

Subjective cognitive decline (SCD) is an early manifestation of Alzheimer’s disease (AD) and offers a therapeutic window where interventions have strong potential to prevent or delay the progression of AD. Aerobic exercise and cognitive training represent two promising interventions for AD prevention, but their synergistic effect has yet to be assessed in persons with SCD.

**Methods/design:**

The purpose of this single-blinded, 3-parallel group randomized controlled trial is to test the synergistic efficacy of an exergame intervention (simultaneous moderate-intensity aerobic cycling and cognitive training) on cognition and aerobic fitness in community-dwelling older adults with SCD. The Exergames Study will randomize 96 participants on a 2:1:1 allocation ratio to 3-month exergame, cycling only, or attention control (stretching). Primary outcomes include global cognition and aerobic fitness, which will be assessed at baseline and after 3 months. The specific aims of the Exergames Study are to (1) determine the efficacy of the exergame in older adults with SCD and (2) assess the distraction effect of exergame on aerobic fitness. Data will be analyzed using ANOVA following intention-to-treat.

**Discussion:**

This study will test the synergistic effects of exergame on cognition and aerobic fitness. It has the potential to advance prevention research for AD by providing effect-size estimates for future trials.

**Trial registration:**

ClinicalTrials.gov NCT04311736. Registered on 17 March 2020.

## Background

Subjective cognitive decline (SCD), the subjective experience of worsening memory or cognitive, is one of the earliest noticeable symptoms of Alzheimer’s disease (AD) [1]. It offers a therapeutic window where interventions have strong potential to prevent or delay the progression of AD because of the increasing recognitions that AD pathology accumulates over years to decades before any observable clinical symptoms [2]. Interventions at later clinical phases of AD have been ineffective or shown only modest benefits [3]. Aerobic exercise and cognitive training are two potential disease-modifying interventions through the induction of brain plasticity and attenuation of AD neurodegeneration [4–6]. Epidemiological studies have linked exercise to reduced risk for AD [7–9]. Likewise, randomized controlled trials (RCT) and meta-analyses of RCTs demonstrate that aerobic exercise produced mild to moderate gains in a wide range of cognitive domains in persons with intact cognition, mild cognitive impairment (MCI), or dementia [10–15]. On the other hand, cognitive training involves repeated practice of a set of standard cognitive tasks targeting specific cognitive domains [16]. Targeting cognitive processes (e.g., attention) may produce more robust and broadly generalizable effects than targeting cognitive structures (e.g., delayed recall) [17, 18]. Previous studies found that such process-based cognitive training generated a moderate to large improvement in selected cognitive domains in older adults with intact cognition or MCI [19–21].

Given that aerobic exercise and cognitive training work through discrete neuronal mechanisms, combined aerobic exercise and cognitive training might have a synergistic and superior effect on cognition compared to either intervention alone [22]. Traditionally, the combined effects of exercise and cognitive training have been investigated in a sequential manner, producing mixed results [6, 23–27]. It is thought that the heterogeneity between the interventions and the delivery of the interventions (i.e., sequentially) may be the primary factors for the inconsistencies [28, 29]. A second interventional delivery option is simultaneous (dual-task) exercise + cognition training (i.e., “exergame”); however, there are limited studies that have investigated this delivery modality [29]. Anderson-Hanley [30] reported results from an exergame intervention (i.e., the “Cybercycle” Study) (cycling + virtual reality path finding and competition with a shadow cycler), a multi-site cluster RCT in which the cognitive benefıt of cybercycling showed superior cognitive effects than traditional stationary cycling, for older adults living independently. More recently, Barcelos [28] compared cycling alone to virtual cycling with a concurrent video game task and found greater effects on cognitive measures from the cycling plus gaming intervention. However, similar studies have not been conducted in older adults with SCD.

### Study aims

The purpose of this phase II RCT is to test the efficacy and additive/synergistic effects of an exergames intervention (simultaneous moderate intensity cycling and cognitive training), in comparison to cycling only and attention control (stretching) on cognition and aerobic fitness in older adults with SCD. The specific aims and hypotheses of the study are described as follows:

- Aim 1: Determine the efficacy of the exergame in older adults with SCD.
- Hypothesis: Exergame will improve global cognition more than cycling only and attention control.
- Aim 2: Assess the distraction effect of an exergame on aerobic fitness.
- Hypothesis 2a: Exergame participants will achieve similar gains in aerobic fitness to cycling participants.
- Hypothesis 2b. Exergame participants will achieve similar exercise intensity targets to cycling participants.

## Methods

### Design

The RCT will use a single-blind, 3-parallel group design, guided by CONSORT [31] and SPIRIT [32] guidelines (*n* = 96). We will use a 2:1:1 allocation ratio to randomize 48 subjects to exergame, 24 to cycling only, and 24 to attention control within age strata (< 75 and ≥ 75 years) and using permuted blocks of 4 and 8 participants. Based on our previous studies, we anticipate screening approximately 288 individuals to enroll 96 participants. Cognition and aerobic fitness will be assessed at baseline and 3 months. This study was approved by the university Institutional Review Board and registered at ClinicalTrials.gov on 17 March 2020 (study identifier NCT04311736).

### Setting

Screening and data collections will occur in a private room at the Clinical and Translational Science Institute or Laboratory of Clinical Physiology at the University of Minnesota, local Young Men’s Christian Association (YMCA) gym, or senior center in close proximity to participant’s residence. The interventions will occur at a local YMCA gym, senior center, or the participant’s residence.

### Study population

#### Recruitment

We plan to enroll 96 participants with a proactive recruitment plan, including presentations at local YMCAs and senior living facility partners, exhibits at relevant conferences, mass email sends, advertisements through social media (i.e., Facebook and Instagram), and recruitment material distributions (i.e., fliers) in the communities.

#### Eligibility and screening

Potential participants who respond to our recruitment strategies and initiate contact with us will be carefully screened for eligibility and exercise safety using a three-step procedure spread over the course of 2–4 weeks, including a phone interview, an in-person interview, and a shuttle walk test (SWT) to ensure they meet our eligibility criteria (Table 1).

1. Phone interview: The staff will receive the respondent’s verbal consent for participating in a 20–30-min phone interview. The phone interview will evaluate the presence of SCD, health history, contraindications to exercise [33], and cognitive status (telephone interview for cognitive status (TICS)) [34]. Potential participants who are not excluded based on the phone screen will be scheduled for an in-person interview.
2. In-person interview: Informed consent, Health Insurance Portability and Accountability Act (HIPAA) authorization, and permission for release of medical records will be obtained by study staff. During informed consent, individuals will be informed about the study procedures, compensation, time commitment, potential risks and benefits, compensations, data collection, the voluntary nature of study participation, and contact within and outside the study team for human subjects. The study staff will corroborate phone screen data; administer questionnaires including Geriatric Depression Scale (GDS) [35], Beck’s Anxiety Inventory (BAI) [36], and Geriatric Anxiety Scale (GAS-10) [37]; and conduct a focused physical assessment. The study coordinator will summarize screening data for review by the investigators to determine eligibility. For eligible participants, letters will be sent to the potential participant’s medical providers to obtain medical clearance for exercise and for excluding the contribution of psychological symptoms to SCD.
3. Shuttle walk test (SWT): Upon receiving exercise safety clearance, participants will complete a SWT to further rule out clinical signs and symptoms indicative of symptomatic cardiovascular disease and to obtain peak heart rate (HR) to be used for purposes of exercise prescription. Resting seated and standing HR and blood pressure will be evaluated to ensure safety prior to performing the test. HR will be continuously monitored during exercise. The test will be stopped if any clinical signs or symptoms develop [33]. HR and blood pressure will be monitored for 6 min following completion of the SWT to ensure residual stability. Those who successfully complete the SWT (i.e., develop no signs and symptoms of symptomatic cardiovascular disease) will be formally enrolled in the study upon completing baseline data collection and subsequently randomized.

**Table 1:** Eligibility criteria

Inclusionary criteria	Exclusionary criteria
a) Subjective Cognitive Decline (SCD: defines as answering yes to the question’s “Do you perceive memory or cognitive difficulties?” and “In the last two years, has your cognition or memory declined”) b) Not engaging in aerobic exercise or cognitive training > 2 days/week, 30 min a session for the past 3 months c) Age 65 years and older d) Ability to provide written consent e) English speaking	a) Resting heart rate > 100 or < 50 beats/min with symptoms b) Dementia or mild cognitive impairment (self-report, diagnosis, or scoring < 26 on the TICS c) Neurological or major psychiatric disorder likely causing SCD d) Alcohol or chemical dependency that likely is causing SCD e) Current enrollment in another intervention study f) ACSM contraindications to exercise or other factors that make exercise impossible or unsafe g) Inability to read due to illiteracy or poor eyesight

*ACSM* American College of Sports Medicine, *TICS* Telephone Instrument for Cognitive Status

#### Sample size and power

Enrollment of 96 subjects with 12% attrition at 3 months will give us 80% power to detect a .85 effect size between exergame and attention control.

### Variables and their measures

#### Primary and secondary outcomes

The primary outcome (Table 2) for this RCT is episodic memory, while secondary outcomes include executive function, global cognition, and aerobic fitness. Outcomes will be assessed at baseline and at 3 months by data collectors who are blinded to group allocation and previously collected data. All cognitive outcomes will be assessed through the NIH Toolbox Cognitive Battery (NIHTB-CB) [38, 39] according to NIH Toolbox Assessment Center instructions [39]. The NIHTB-CB, an interactive personal application device (iPAD) based battery of executive function, attention, episodic memory, language, processing speed, and working memory tests, was developed within the NIH Blueprint for Neuroscience Research and has been validated and normed in a broad sample of the US population [38, 39]. This battery yields summary scores such as the Cognitive Functioning Composite Score, in addition to individual measure scores as outlined below [38, 39].

**Table 2:** Data collected from participants during the study

Variables	Measures (data type)	Data collection type	Screening period	Baseline/follow-up period
Cognitive outcomes				
Episodic memory	Picture Sequence Memory Test, Auditory Verbal Learning Test (both continuous)	NIH toolbox (iPAD)		x
Executive function	Cognitive Battery Flanker Inhibitory Control, Attention Test and Dimensional Change Card Sort Test (both continuous)	NIH toolbox (iPAD)		x
Global cognition	Composite score from all tests for discrete cognitive domains	NIH toolbox (iPAD)		x
Aerobic fitness outcomes				
Aerobic fitness	SWT (continuous)	Performance-based test		x
Other variables				
Depression	GDS (continuous)		x	
Anxiety	(Gas-10), BAI (both continuous)		x	
Physical function	SPPB, 6MWT (both continuous)	Performance-based test		x
Medical change	Medical diagnoses, falls, medications (all categorical)		Monthly	
Demographics	Age, education (both continuous) sex, race (both discrete)	Self-report, paper form	x	
Physical activity	Accelerometry, PASE questionnaire (both continuous)	Wrist device, pencil-paper-based questionnaire	Monthly	
Intervention adherence	Percent (continuous)	REDcap report		
Usability, acceptability, and satisfaction of exergame	Likert scale (ordinal)	Pencil-paper-based questionnaire	Monthly	

6MWT 6-min walk test, BAI Beck’s Anxiety Inventory, Gas-10 Geriatric Anxiety Scale – 10 item, GDS Geriatric Depression Scale, PASE Physical Activity Scale for the Elderly, SWT shuttle walk test

#### Episodic memory

Episodic memory refers to cognitive processes involved in the acquisition, storage, and retrieval of new information and will be measured using the Picture Sequence Memory Test (PSMT) [39] and Auditory Verbal Learning Test (Rey) (RAVLT) [39] (supplementary measure). The PSMT has excellent test-retest reliability (*r* = 0.84) and is strongly correlated with the RAVLT, (*r* = .64), reflecting good convergent validity [40].

#### Executive function

Executive function refers to the capacity to plan, organize, and monitor the execution of goal-directed behaviors such as set-shifting and inhibitory control and will be measured by the Flanker Inhibitory Control and Attention Test [39] and Dimensional Change Card Sort Test [39]. Both tests demonstrate excellent sensitivity to age-related changes during adulthood, excellent test–retest reliability (*r* = .85), and adequate to good convergent validity (*r* = .52–.55) [41].

#### Global cognition

Global cognition refers to the overall cognitive ability and will be calculated as the NIHTB-CB Cognitive Functioning Composite Score (i.e., total score). The Cognitive Functioning Composite Score is calculated from the following domain-specific scores: language (Picture Vocabulary Test and Oral Reading Recognition Test), attention (Flanker Inhibitory Control and Attention Test), processing speed (Pattern Comparison Processing Speed Test and Oral Symbol Digit Test [supplementary measure]), and working memory (List Sorting Working Memory Test) in addition to the tests used to measure episodic memory and executive function. The composite score has high psychometric measurements including internal consistency (Cronbach’s alpha = .77), test-retest reliability (*r* = .90), and convergent validity (*r* = .89) in adults [42].

#### Aerobic fitness

Aerobic fitness will be assessed using the SWT, following the guidelines by Singh and colleagues [43]. Participants will be required to walk along a level, 10-m course at a previously determined speed dictated by signals from an audio recording. Laps will be counted for calculation of peak walking distance achieved during the SWT. The test will be considered finished when the participant is not able to maintain the required speed (more than 0.5 m from the cone) or for some other reported symptom that warrants test termination (i.e., American College of Sports Medicine [ACSM] clinical sign or symptom for exercise test termination) [33]. Because older adults with SCD may have trouble multitasking and remembering test instructions, we will provide additional instructions to ensure proper testing methodology including by providing additional verbal instruction including (1) “stop” when participants arrive at the marker before the beep, (2) “go” when the single beep for walking sounds, and (3) “go and walk faster now” when the triple beep sounds, indicating faster pace required [44]. The SWT reliability is well established in older adults with multiple chronic conditions (interclass correlation 0.91–.97) [45, 46]. Additionally, the peak walking distance on SWT is highly correlated to peak oxygen consumption derived from cardiopulmonary exercise testing in older adults with multiple chronic conditions [47], making it a valid test for assessing aerobic fitness.

### Study procedure

#### Study preparation and randomization

All staff will be adequately trained to ensure participant safety, blinding, data quality, and protocol adherence based on the Study Manual of Operations. Prior to any recruitment, the statistician will create a randomization schedule in Research Electronic Data Capture (REDCap). Qualified participants will be randomized after completing baseline data collection. The study coordinator will log into the REDCap randomization module and enter a participant’s name/age. The randomization module will assign the participant to a group and record the assignment in a database that is only accessible to unblinded staff.

#### Data collection

Enrolled subjects will be scheduled to complete baseline data collection. Subsequent data collection will occur at 3 months. Data collectors will be independent from study interventionists to help insure data integrity. To ensure the blindness of the data collectors from the intervention assignment, the data collectors will not interact with enrolled subjects except for data collection.

#### Delivery of the assigned activity for each group

Within 2 weeks of completing baseline data collection, participants will start their assigned activity: exergames, cycling only, or attention control. Each activity will consist of a total of three weekly, supervised sessions for 3 months (36 total sessions). One interventionist will supervise up to three participants in a single visit (1:3 interventionist/participant ratio) in the same activity. The sessions will be delivered over up to a 14-week period to account for missing sessions due to vacations and illnesses.

#### Cycling group

Subjects will cycle on recumbent stationary cycles at moderate intensity individualized as 50–70% of heart rate reserve (HRR) and/or 11–14 on Borg Category Ratio-15 Rating of Perceived Exertion (RPE) Scale. HRR will be calculated by subtracting resting HR (after 10-min quiet resting) from the peak HR achieved during the SWT. Cycling will be progressed from 50–60% of HRR or RPE 11–12 for 30–35 min in session 1 and will be alternatively increased by 5% of HRR (or 1-point on Borg) or 5-min increments as tolerated up to 60–70% of HRR (or RPE 12–14) for 50 min a session over time. All sessions will include a 5-min warm-up and 5 cool-down before and after cycling, in addition to the prescribed exercise duration, following the ACSM guidelines [33]. In each session, the interventionists will help subjects put on a Polar™ wireless HR monitor (RS400, Lake Success, NY), take resting HR and blood pressure, and monitor HR, RPE, talk test, and signs and symptoms every 5 min, and blood pressure every 15 min.

#### Exergame

Subjects will cycle as described in the cycling protocol while engaging in cognitive training for the duration of cycling. Cognitive training will include one level of difficulty with six task scenarios in the context of three virtual worlds (environments): “Small-Town Downtown,” “Underwater World,” and “Wild West.” For example, the virtual world of “Small-Town Downtown” will be composed of shops, restaurants, storefronts, and office buildings. Each environment will be open-world and static and will not change over time or between game sessions. This will allow the participant to memorize the layout over multiple play sessions. Visual cues (“Landmarks”) will serve as memorization guides for navigation. Landmark examples for “Small-Town Downtown” include unique buildings, bridges, statues, parks, and other true-to-the-environment objects. Scenarios will have high ecological validity. Scenario examples in “Small-Town Downtown” include (1) picking up and dropping off a delivery for a friend, (2) visiting the post office to mail a letter, (3) sorting books at the library, and (4) going shopping at the grocery store. In each of the environments, the participant must follow directions and navigate to a destination where the cognitive task will be performed. For each scenario, participant start position within the environment will be randomly assigned by the system. A scenario-specific cognitive task will be triggered. The game will inform the participant of the assigned task and, automatically, determine and inform the participant of the fastest route from the starting point to the scenario destination where the task will be performed. The participant will be required to remember the assigned task, follow the directions and navigate to the destination, and then remember what action is required to complete the task. The participant will move forward through the environment by pedaling the stationary cycle. Left and right turns along the route will be input by the participant through buttons on the game controller. To enable a smooth transition into the cognitive task while maintaining cycling movement, the game’s camera will continue to progress in a forward direction to match participant pedaling speed, while the level environment fades into a desaturated surreal version of each scenario’s setting.

#### Attention control

Participants will participate in passive stretching that we have previously tested [48, 49] and that are matched to session durations for cycling and exergame groups. Light intensity (RPE ≤ 9 or HRR ≤ 30%) stretching exercises include seated movements and static stretches that induce no changes in aerobic fitness.

#### Treatment fidelity

This study was designed to ensure treatment fidelity based on the NIH Behavior Change Consortium recommendations [50] and our experiences [48, 49]. The investigators and the study coordinator will oversee adherence to the protocol by all staff through review focused on screening compliance, study outcomes, intervention compliance (i.e., percent of exercise sessions attended, percentage of session duration in target intensity range, and exercise dose), and adverse events. Investigators and the study coordinator will ensure data creation and completeness through quality check and audits. Weekly meetings will be held among the investigators and study staff to ensure compliance to study protocol, while monthly fidelity checks will be performed (total 3% of intervention sessions) by the investigators to ensure accurate implementation of intervention protocols by using a previously used Fidelity Checklist [48]. Staff will be retrained as needed. The investigators will review 3% of session case forms (i.e., exercise logs) to assess for protocol drift and completeness.

#### Safety, retention, adherence, and validation

We have built in many strategies that have been successfully utilized in our preliminary studies to protect participants against risks, including ongoing monitoring of health status changes, appropriate equipment and personnel training, individualized prescriptions and supervised delivery for cycling and exergames training, and careful screening and informed consent. Interventionists will be fully trained using the Study Manual of Operations and will be able to consult with the investigators on an immediate, as-needed basis as well as at weekly meetings. During the course of the intervention, if a participant develops a contraindication to exercise, interventions will be terminated until the participant is re-cleared to resume exercise by their primary care provider or cardiologist.

### Data management

#### Data entry, coding, and storage

All participant data will be identified by a screening identification (ID) and a study ID for enrolled participants. Access to the links between name and IDs will be restricted to the study coordinator and interventionists involved in screening. Data collection forms returned to the research office by the study staff will be reviewed by the study coordinator for accuracy and completeness before entry into the study REDCap database [51, 52]. Data collection forms will then be stored in a specific office that is further locked and contains a lockable storage cabinet specific for data collection storage. Cognitive data from the NIH Toolbox Cognitive Battery will be exported from study iPAD into a university email account and then subsequently uploaded into REDCap by data collectors. Additionally, the study coordinator will verify data accuracy and completeness in REDCap. Any missing data will be assigned to a data collector for collection. If a participant withdraws from the study, all attempts will be made to collect data to allow for inclusion in the analysis. Reasons for withdrawal will be recorded. Data audits of electronic outcome data will be conducted by the study coordinator. If any discrepancies in scoring between the study staff who collected data and the study staff who entered data, a meeting with the investigators or study coordinator and involved study staff will be undertaken to ensure the correct data is in REDCap.

#### Statistical analyses

All variables will be summarized using appropriate descriptive statistics (e.g., means/standard deviation [SD] for continuous measures, and frequencies for categorical variables). Distributions will be visually inspected for outliers, and determine the best link and distribution combination for each outcome using the quasi information criteria [53]. Statistical assumptions will be checked. Baseline variables will be compared among the 3 groups to determine systematic differences using Pearson’s chi-square test (categorical variables) and one-way analysis of variance (ANOVA) or Kruskal-Wallis (continuous variables), as appropriate. Analysis will be accomplished using Statistical Product and Service Solutions (SPSS) for Windows or Statistical Analysis System (SAS). Variables that differ significantly among groups will be included as covariates in the models described below. All analyses will follow the intention-to-treat principle.

- Aim 1: Determine the efficacy of the exergame in older adults with SCD. To test our hypothesis that exergame will improve global cognition more than cycling only and attention control, the ANOVA will be performed. If statistically significant results are identified, then we will perform post hoc analysis to determine how the changes in cognition differ between groups. If homogeneity of variances are supported across the three groups, we will conduct Tukey’s honestly significant different post hoc test. If homogeneity of variances are violated, then we will perform the Games Howell post hoc test. If significant covariates are identified, then analysis of covariance (ANCOVA) will be used instead. Linear simple and multiple regressions will be performed to assess association between the effects of exergames on global cognition and aerobic fitness. A *p* value < 0.05 will be used to determine statistical significance. Effect size (EF) calculated as EF = (mean exergames group − mean stretching group)/SD (stretching group) will be used to assess the clinical significance of the effects of exergames on cognitive and aerobic fitness outcomes listed above.
- Aim 2: Assess the distraction effect of exergame on aerobic fitness. Our hypothesis that exergame participants will achieve similar gains in aerobic fitness to cycling participants will be analyzed similarly to aim 1 hypothesis using ANOVA and post hoc test. In addition, heart rate monitor data will be utilized to compute intensity dose, to compare objective effort given during exergame and cycling only sessions. We hypothesize that the average %HRR achieved by exergame participants will not be significantly different compared to cycling only participants. A standard formula will be used to quantify the intensity dose and is as follows. Percent HRR (%HRR) averaged in each session will be calculated from the following variables (EHR = exercise session average heart rate, PHR = peak heart rate from SWT, and RHR = resting heart rate from SWT) and formula: %HRR = [(EHR − RHR)]/(PHR − RHR)]. The %HRR will be averaged across all sessions to give a quantifiable index of intensity to be used as a surrogate measure of distraction. Differences between exergames and cycling only groups on average %HRR will be assessed by independent *T* test.

### Ethics

This study was approved by the university Institutional Review Board (IRB), and any amendments or subsequent changes or to the protocol will be submitted to the IRB for approval. Written consent takes place during the in-person interview. Participant’s capacity to consent will be assessed by staff through use of the UCSD Brief Assessment of Capacity to Consent form (UBACC) [54] modified for exergame. A participant must score a “2” on items 1, 2, 4, 6, 7, and 9 on the assessment for inclusion in the study. If a participant scores less than 2 on any of these items, the staff will re-explain the study, and then ask the participant to return on another day to retake the UBACC. If the participant scores < 2 on any of these items again, then the subject is not eligible to participate in the study. Consenting will be an ongoing process during the study as verbal consent will be obtained at the beginning of each activity session. Participation in exercise is known to cause musculoskeletal pain or discomfort during exercise or 24–48 h following exercise (due to commonly seen delayed onset muscle soreness). Study-related, anticipated adverse events such as these will not be reported to IRB; however, all study-related unanticipated adverse events (such as cardiopulmonary events) will be reported to the IRB and funding agency within 5 business days of the event.

### Dissemination

Findings from the RCT will be disseminated through presentations and publications. Participants and their family members will receive a copy of the published main findings. All manuscripts will be authored by the study team and authorship will follow the established publication guidelines such as those of the International Committee of Medical Journal Editors. Access to the final trial data set will follow the guidelines of the National Institute on Aging.

## Discussion

Developing interventions, with strong potential to prevent or delay the onset of AD in persons with SCD is critical. Aerobic exercise and cognitive training represent two promising interventions; however, their synergistic effects on preventing AD have not been evaluated. Our study will develop a novel, apple-TV, and iPad-based cognitive training that is immersive, naturalistic, and similar in tasks to real life, and therefore increases the ecologic validity of the treatment [29]. It is cognitively challenging but also increases exercise enjoyment.

All attempts have been undertaken to ensure the highest quality of study design and delivery of the Exergames Study. First, RCTs are considered the gold standard design for determining the causal effect of an intervention on an outcome. Alternatively, intervention trials commonly employ usual care or waitlist control groups or cross-over design. Usual-care or waitlist controls preclude assessing the Hawthorne or placebo effect of exergames, while cross-over designs are ideal for interventions without carry-over effects, which is also not the case for exergames. In addition, we have built in four strategies to prevent unblinding, another potential problem occurring in RCTs. The four unblinding prevention strategies include (1) assessors will not interact with other staff who are not blinded (i.e., exercise interventionists) and will participate in separate meetings, (2) investigators will be blinded to group assignments, (3) participants will be blinded to the study aims and reminded as needed not to discuss their experiences with the outcome assessor, and (4) randomization will be completed using permuted blocks. Additionally, age is a known, strong factor affecting cognition. While our aims are not focused on age, we will stratify enrollment by age to equalize age distribution across groups [55, 56].

In summary, the Exergames Study will be the first RCT to examine the additive/synergistic effects of a novel simultaneous moderate intensity, supervised aerobic exercise + cognitive training intervention on cognition and aerobic fitness. This study looks to advance AD prevention research by providing precise immediate and long-term effect-size estimates of an aerobic exercise intervention delivered with simultaneous cognitive training to inform future fully powered, large scale phase III RCTs.

## Trial status

ClinicalTrials.gov identifier NCT04011267, version 1.0, 17 March 2020. Registered on 17 March 2020 (reterospectively registered) and at the time of manuscript submission has not been updated. The status of the trial at the time of manuscript submission is open for enrollment. Participant enrollment began on 27 December 2019 and we expect enrollment accrual to complete in 2022.

## Data Availability

Not applicable

## Abbreviations

ACSM: American College of Sports Medicine
AD: Alzheimer’s disease
ANCOVA: Analysis of covariance
BAI: Beck’s Anxiety Inventory
CONSORT: Consolidated Standards of Reporting Trials
EF: Effect size
Gas-10: Geriatric Anxiety Scale – 10-item version
HIPPA: Health Insurance Portability and Accountability Act
HR: Heart rate
HRR: Heart rate reserve
ID: Identification
iPAD: Interactive personal application device
IRB: Institutional review board
MCI: Mild cognitive impairment
NIHTB-CB: NIH Toolbox Cognitive Battery
PSMT: Picture Sequence Memory Test
RAVLT: Auditory Verbal Learning Test (Rey)
RCT: Randomized controlled trial
REDcap: Research Electronic Data Capture
RPE: Rating of perceived exertion
SAS: Statistical Analysis System
SCD: Subjective cognitive decline
SPIRIT: Standard Protocol Items: Recommendations for Interventional Trials
SPSS: Statistical Product and Service Solutions
SWT: Shuttle walk test
UBACC: USCD Brief Assessment of Capacity to Consent
VRCT: Virtual reality cognitive training
YMCA: Young Men’s Christian Association
